# Origin-Oriented Elemental Profile of Fine Ambient Particulate Matter in Central European Suburban Conditions

**DOI:** 10.3390/ijerph13070715

**Published:** 2016-07-15

**Authors:** Wioletta Rogula-Kozłowska, Grzegorz Majewski, Barbara Błaszczak, Krzysztof Klejnowski, Patrycja Rogula-Kopiec

**Affiliations:** 1 Polish Academy of Sciences, Institute of Environmental Engineering, M. Skłodowskiej-Curie 34, Zabrze 41-819, Poland; barbara.blaszczak@ipis.zabrze.pl (B.B.); krzysztof.klejnowski@ipis.zabrze.pl (K.K.); patrycja.rogula-kopiec@ipis.zabrze.pl (P.R.-K.); 2 Faculty of Civil and Environmental Engineering, Warsaw University of Life Sciences, Nowoursynowska 166, Warszawa 02-776, Poland; grzegorz_majewski@sggw.pl

**Keywords:** respirable particulates, source apportionment, MLRA, PCA, traffic emissions, municipal emission, coal combustion, hotspot area in Europe

## Abstract

Twenty-four-hour samples of fine ambient particulate matter (PM_2.5_; particles with aerodynamic diameters ≤2.5 µm) were collected in a suburban (quasi-rural) area in Racibórz (Poland) between 1 January 2011 and 26 December 2012. The samples were analyzed for the contents of 28 elements. Sources of PM_2.5_ were identified and the contribution of each source to the PM_2.5_ concentration was assessed using an enrichment factor (EF) analysis, a principal component analysis (PCA), and multi-linear regression analysis (MLRA). In the cold season (January–March and October–December 2011–2012), the mean ambient concentration of PM_2.5_ in Racibórz was 48.7 ± 39.4 µg·m^−3^, which was much higher than at other suburban or rural sites in Europe. Additionally the ambient concentrations of some toxic PM_2.5_-bound elements were also high, i.e., the mean ambient concentrations of PM_2.5_-bound As, Cd, and Pb were 11.3 ± 11.5, 5.2 ± 2.5, and 34.0 ± 34.2 ng·m^−3^, respectively. In the warm season (April–September 2011–2012), the PM_2.5_ and PM_2.5_-bound element concentrations in Racibórz were comparable to the concentrations noted at other suburban (or rural) sites in Europe. Our findings suggest that elemental composition and concentrations of PM_2.5_ in Racibórz are mainly influenced by anthropogenic emissions, i.e., the energy production based on coal and biomass combustion, traffic, and industry.

## 1. Introduction

Among all air pollutants, particulate matter (PM) affects the environment most extensively. It contributes to a variety of health effects and climate changes [1,2,3,4,5,6,7,8,9]. The strength and direction of this influence depends on the concentration of PM and its physicochemical properties. Therefore, the most sophisticated apparatus and methods are used to investigate the chemical composition of PM. Knowledge of the chemical composition of PM is fundamental to enable proper identification of PM sources. It also reveals the PM source-receptor links. Moreover, it allows researchers to develop more efficient PM emission reduction methods. The relationships between the elemental composition and the origin of PM have been intensively studied for the last ten years. One of the most common receptor modelling techniques [10,11,12,13,14] used for assessing the origin of PM based on its elemental composition is principal component analysis (PCA) combined with multi-linear regression analysis (MLRA) (Table 1). Despite significant progress in the identification of PM pollution sources, knowledge of the possible origin of PM_2.5_ (fine particles; aerodynamic diameters ≤2.5 µm) is still incomplete. There is a limited number of appropriate PM_2.5_ sampling points in Central and Eastern Europe (Table 1), which substantially restricts accurate identification of PM sources [15]. 

An interesting contrast can be noticed between the origin of ambient particulate matter in Western Europe and in Poland, especially in the highly urbanized and industrialized Silesia region (one European PM pollution hotspot), where municipal and industrial emissions and emissions from energy production are greater than traffic emissions. The structure of air pollution in Poland is representative of Eastern and Middle Europe countries, where the energy production relies on the combustion of fossil fuels (mainly coal). Zabrze and Katowice (Poland) are well monitored areas in terms of PM concentrations and elemental composition of PM_2.5_. The two cities, located approximate 60 km north of Racibórz, are typical in terms of atmospheric air pollution by PM. Both in Zabrze and in Katowice very high mass contributions of PM_2.5_ in total PM are observed, particularly in winter [16,17,18,19]. Analysis of the chemical composition of fine PM suggests anthropogenic sources of PM (domestic furnaces, fireplaces, road traffic, etc.) [19,20,21].

In European rural areas most of PM comes from long-distant pollutant transport and natural sources. Its concentration is generally lower than in city centers and rarely causes any air quality problems [57,58,59,60,61,62,63]. However, there are suburban (quasi-rural) areas, such as Racibórz, which are neither “urban” (i.e., they are neither densely populated nor built up and the PM origin differs from that in urban areas) nor “rural” (being medium-size towns which are as common in southern Poland as industrial towns and cities like Zabrze or Katowice). To our knowledge, long-term observation of elemental composition of PM_2.5_ has not been carried out in any suburban areas located in a European hotspot yet. Still, some research shows that PM–related exposure among Racibórz residents is the same as or sometimes even higher than that found among the inhabitants of city centers [21]. 

The purpose of this work is to identify the souces of PM_2.5_ in a suburban area located in southern Poland, based on statistical analysis of a two-year data series of elemental composition of PM_2.5_.

## 2. Materials and Methods

### 2.1. PM_2.5_ Sampling

Twenty-four-hour PM_2.5_ samples were collected by means of a PNS (peripheral nervous system) Atmoservice sampler (Poznań, Poland) equipped with a PM_2.5_ head (PM inlet with PM_2.5_ sharp-cut cyclone). The air flow rate was 2.3 m^3^·h^−1^. The PM_2.5_ samples were collected on 47-mm diameter PTFE membranes (Whatman Cat. No. 7592-104, with a minimum particle filtration effectiveness of >0.2 μm diameter equal to 99.95%. Little Chalfton, UK). Before and after the exposure, the filters were conditioned in a weighing room (48 h; relative air humidity: 45% ± 5%; air temperature: 20 ± 2 °C) and weighed twice (at 24 h intervals) on a Mettler Toledo AT microbalance (with a resolution of 2 µg) using a Haug U-ionizer (Mettler Toledo, Warsaw, Poland). The adopted method of PM sampling and the subsequent gravimetric analysis of the PM were compliant with EN 14907/2005 (Standard gravimetric measurement method for the determination of the PM_2.5_ mass fraction of suspended particulate matter) and EN 12341/1998 (Air quality-Determination of the PM_10_ fraction of suspended particulate matter-reference method and field test procedure to demonstrate reference equivalence of measurement methods). The weighing accuracy, determined as three standard deviations from the mean obtained from ten weighings of a blank filter (conditioning performed every 48 h), was 20.5 µg.

### 2.2. Sampling Site

**Figure 1:**
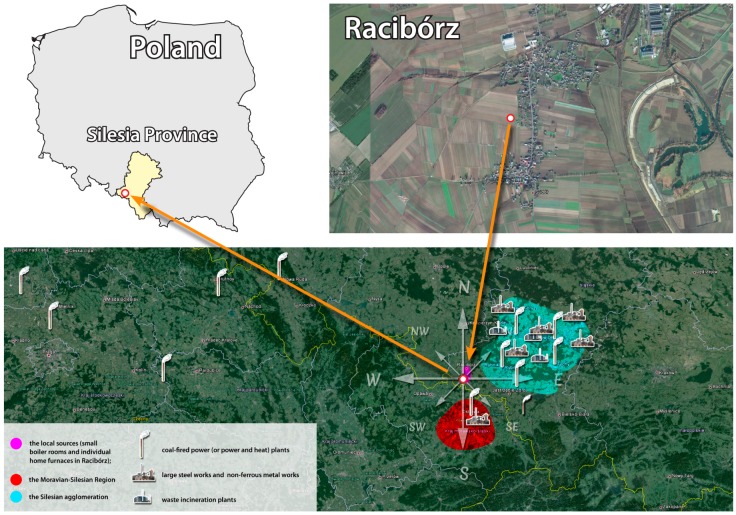
Sampling point location.

The samples were collected between 1 January 2011 and 26 December 2012 in Racibórz, a town located in the south-western part of the Śląskie (Silesian) Province in south Poland, close to the Czech-Polish border. The sampling point (50°5′ N and 18°14′ E) was located approximate 6 km south of the center of Racibórz. The Racibórz area is an agricultural region (Figure 1), where farmlands cover approximate 66.30% of the area. The entire industry (a few chemical, power, agricultural, and food-processing plants) as well as commercial centers are located in the town center. The population density is approximate 220 people/km^2^ in the whole area, and about 700 people/km^2^ in Racibórz itself. It is 3–5 times less than in other cities and towns in southern Poland. The majority of the inhabitants live in Racibórz. Low buildings with individual heating systems (fed with biomass, coal, or natural gas) dominate the landscape. The building development is compact in the center of Racibórz and scattered in the vicinity of the sampling site. The mean annual temperature in the region is +8.0 °C. January is the coldest month (−2.1 °C), whereas July is the warmest one (+18.0 °C). During the year, there are 100–110 frost days and 170 days with precipitation (including 45 snowy days) in the region. South winds prevail in autumn and winter and north winds are common in spring and summer. Calms are frequent.

### 2.3. Elemental Analysis

The PM_2.5_ samples (320 in total) were analyzed for their elemental content by means of energy dispersive X-ray fluorescence (EDXRF) on an Epsilon 5 spectrometer (PANalytical B.V.; Almelo, The Netherlands). The apparatus was equipped with an X-ray tube with a side window (nitrogen-cooled, gadolinium anode, working range 25–100 kV, 150-μm beryllium window), a system of nine secondary targets (Al, Ti, Fe, Ge, Zr, Mo, Ag, Ce_2_O_3_, Al_2_O_3_), and a Ge(Li) detector (resolution 140 eV, energy range 0.7–100 keV, working surface 30 mm^2^, 8-μm beryllium window). The measurements were conducted under vacuum condition. The analysis, including the run of the whole program of the changes of targets and X-ray tube settings (25 keV and 25 mA for Al, 40 keV and 15 mA for Ti, 40 keV and 15 mA for Fe, 75 keV and 8 mA for Ge, 100 keV and 6 mA for Zr, 100 keV and 6 mA for Al_2_O_3_) lasted 2400 s. The concentrations of particular analytes were derived by comparing the results with calibration curves. The curves were determined by measuring thin-layer standards (Micromatter, Inc., Vancouver, BC, Canada) and corrected for possible matrix effects according to a standard procedure saved in the spectrometer software.

The NIST standard reference material (SRM2873) was measured weekly. Except 52% and 39% recoveries of V and Co, the recovery of each element was between 85% (As) and 120% (Pb) of the certified value. Ten blanks (PTFE membranes) were used to determine the detection limits for the procedure. Each blank underwent the entire EDXRF procedure devised for a regular sample 30 times; a detection limit for an element was the standard deviation from the 300 results obtained for this element. The detection limits were between 0.2 (Se) and 21.3 (Si) ng·cm^−2^.

### 2.4. Results Analysis

Twenty-four-hour concentrations of PM_2.5_ and twenty-four-hour PM_2.5_-bound elements were divided at first into two sets and averaged. The measurement period was used as the division criterion. The results were therefore averaged for two periods: the cold/heating season (January–March and October–December, *n* = 147) and the warm/non-heating season (April–September, *n* = 173). A nonparametric technique known as the Mann-Whitney U test was used to test the significance of inter-season variation in PM_2.5_ and PM_2.5_-bound element concentrations (Table 2). The Mann-Whitney U test is a nonparametric test of the null hypothesis, that two samples come from the same population, against an alternative hypothesis, that a particular population tends to have larger values than the other. The Mann-Whitney U test is a non-parametric test, hence it does not assume any assumptions related to the distribution. 

**Figure 2:**
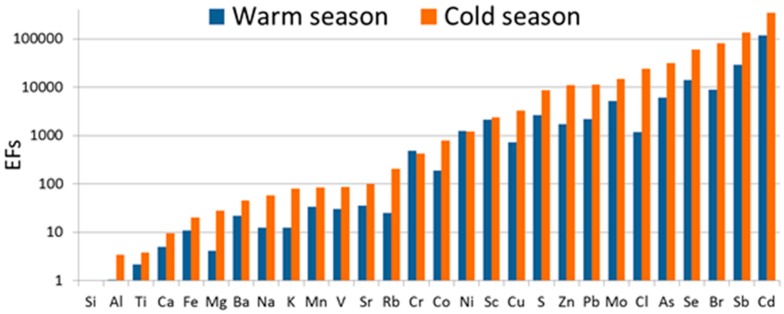
The enrichment factors (EF) for the elements in PM_2.5_ averaged for the cold/heating season (January–March and October–December 2011–2012) and the warm/non-heating season (April–September 2011–2012) in Racibórz.

The so-called enrichment factor (EF, Figure 2) was then calculated for each element measured independently in the heating and the non-heating period. By using the enrichment factor it was possible to assess quantitatively the strength of the anthropogenic effect influencing the PM_2.5_-bound element concentrations [16,18,19,20,21]. 

The enrichment factor *EF_x_* is defined for the element *x* as:

(1) $$EF_{x}=\frac{{\left(C_{x}/C_{ref}\right)}_{PM}}{{\left(C_{x}/C_{ref}\right)}_{soil}}$$

where: *C_x_* and *C_ref_* designate the element *x* and the reference element concentrations, respectively; (*C_x_*/*C_ref_*)*_PM_* and (*C_x_*/*C_ref_*)*_soil_* are proportions of these concentrations in PM and in the soil. 

In the paper, the observed *C_x_* concentrations are related to the *C_Si_* concentration of Si. Consequently, *EF_Si_* = 1. The element concentrations of the Earth’s upper continental crust soil were taken from the study [64]. 

In this study, an *EF_x_* with a value up to 10 indicates the crustal origin of the element *x* [16,18,19,20,21], while a higher *EF_x_* suggests a strong anthropogenic effect on the concentrations of *x*. 

Further data analysis consisted in applying the methodology described by Thurston and Spengler [65]. The method combines a factor analysis (FA) for identifying the possible sources with a multi-linear regression analysis (MLRA) used to quantify their contribution to the PM concentrations. 

In the first step, a principal component analysis (PCA) was applied to the 28 × 320 data matrix representing the twenty-four-hour PM_2.5_-bound element concentrations. Subsequently, values of the new variables—principal components (only principal components with eigenvalues >1.0 were considered according to the Kaiser criterion)—were used in the MLRA. All the calculations were performed using Statistica 8.0 software (StatSoft, Tulsa, OK, USA).

## 3. Results and Discussion

### 3.1. Concentrations of PM_2.5_

The twenty-four-hour PM_2.5_ concentrations in Racibórz ranged from 3.2 µg·m^−3^ to 209.5 µg·m^−3^ (Table 2). The mean PM_2.5_ concentration for the entire measurement period was equal to 29.9 µg·m^−3^. It was relatively high when compared to the PM_2.5_ concentration limits established by the European Commission (25 µg·m^−3^; annual mean value) [66] and the World Health Organization (10 µg·m^−3^; annual mean value) [67]. 

**Figure 3:**
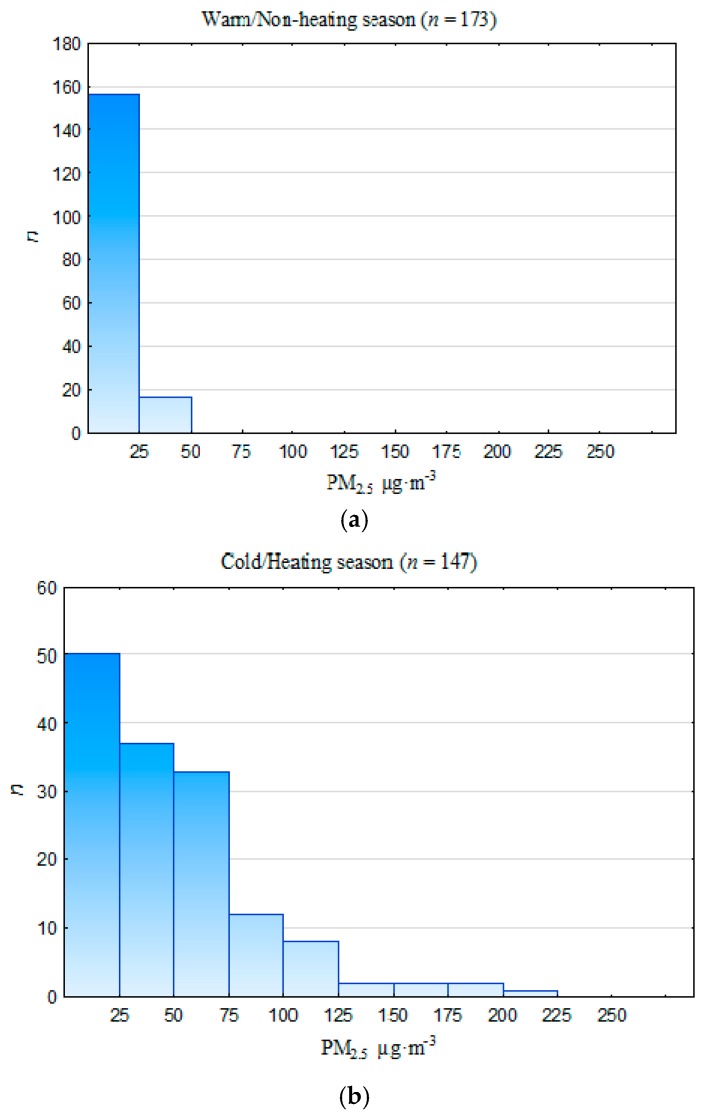
Number of incidents of 24-h concentrations of PM_2.5_ within the assumed ranges for (**a**) the cold/heating season (January–March and October–December 2011–2012) and (**b**) the warm/non-heating season (April–September 2011–2012) in Racibórz.

The twenty-four-hour PM_2.5_ concentrations clearly indicate a poor quality of air as far as pollution with fine PM in the cold (heating) season in Racibórz is concerned (Figure 3). Only 34% of the twenty-four-hour PM_2.5_ concentrations during the cold season in 2011–2012 were below 25 µg·m^−3^, whereas more than 35% of the twenty-four-hour values of PM_2.5_ concentration exceeded 50 µg·m^−3^. During the warm (non-heating) season in 2011–2012, less than 11% of the twenty-four-hour PM_2.5_ concentrations exceeded 25 µg·m^−3^. A distinct seasonality of the concentration of PM_2.5_ and other air pollutants has already been observed in other parts of Poland [68,69,70] and throughout Europe [25,57,63,71]. The seasonal pattern in the PM_2.5_ concentrations was characterized by a maximum in the cold period and a minimum in the warm period (Table 2). The non-parametric Mann-Whitney U test confirmed that the differences between the mean PM_2.5_ concentrations noted in the cold and warm season (Table 2) were statistically significant (α = 0.05; *p* ≤ 0.001); the mean PM_2.5_ concentration in the cold season (48.7 µg·m^−3^) was about 3.5 times higher in comparison with the warm season (13.9 µg·m^−3^).

In the cold season, Racibórz residents increased combustion activities for heating purposes, which generally contributed to the increased PM_2.5_ concentrations [21,70]. In winter, low temperature may have also resulted in a low inversion layer with more PM trapped near the ground level [72,73]. The low twenty-four-hour PM concentrations in the warm (non-heating) season were attributed to the wet removal of aerosol particles (due to more frequent and more abundant precipitation in the warm season than in the cold one) and higher mixing height facilitating the PM dilution and dispersion [48,70]. In Racibórz, where no large-point emission sources exist, the seasonal variations in the PM_2.5_ concentrations were also affected by the incoming air masses from distant areas [21,70].

In 2011–2012, the mean PM_2.5_ concentration in Racibórz did not differ much from the PM_2.5_ concentrations recorded in other cities of southern Poland such as Zabrze (2007; PM_2.5_ = 22.0 µg·m^−3^), Katowice (2007; PM_2.5_ = 31.0 µg·m^−3^) [16], Wrocław (2012; PM_2.5_ = 36.0 µg·m^−3^) [23], Trzebinia (2013; PM_2.5_ = 25.2 µg·m^−3^) and Złoty Potok (2013, PM_2.5_ = 25.2 µg·m^−3^) [74]. This concentration was relatively high as compared to the PM_2.5_ concentrations in other parts of the country, such as Diabla Góra (2010; PM_2.5_ = 15.0 µg·m^−3^) [70] and Szczecin (2010; PM_2.5_ = 17.1 µg·m^−3^) [74] in northern Poland, or Zielonka (2011; PM_2.5_ = 16.1 µg·m^−3^) [75] and Warsaw (2013; PM_2.5_ = 10.7 µg·m^−3^) in central Poland [15]. The mean PM_2.5_ concentration in Racibórz was also high as compared to the values registered at various European stations situated in suburbs, such as Košetice (Czech Republic, 2009–2010, PM_2.5_ = 15.7 µg·m^−3^) [63], Hyytiälä (Finland; 2011, PM_2.5_ = 5.1 µg·m^−3^), Waldhof (Germany; 2012, PM_2.5_ = 11.5 µg·m^−3^), Harwell (UK; 2012, PM_2.5_ = 12.8 µg·m^−3^), Cabauw-Zijdeweg (The Netherlands; 2011, PM_2.5_ = 15.3 µg·m^−3^) and Ispra (Italy; 2010, PM_2.5_ = 17.9 µg·m^−3^) [75]. It was more similar to the PM_2.5_ concentrations registered at industrial sites (e.g., Bobadela (Spain), 2001, PM_2.5_ = 24.0 µg·m^−3^) [35] or traffic-exposed sites (e.g., Athens (Greece), 2007, PM_2.5_ = 35.9 µg·m^−3^) in Europe [76].

### 3.2. PM_2.5_-Bound Elements 

The mean concentrations of the selected PM_2.5_-bound elements ranged from 0.8 ng·m^−3^ (Se) to 2364.3 ng·m^−3^ (Cl) and from 0.3 ng·m^−3^ (Se) to 924.4 ng·m^−3^ (S) in the cold and the warm seasons (2011–2012), respectively (Table 2). The masses of 28 PM-bound elements collectively accounted for 10.4% (cold season) and 13.9% (warm season) of the PM_2.5_ mass. Cl and S were the most abundant among the determined elements; their common percentage in PM_2.5_ was ~8.0% in both measurement periods.

The mean concentrations of the PM_2.5_-bound Cl (1197.6 ng·m^−3^) and S (1201.7 ng·m^−3^) in Racibórz (averaged for the entire measurement period) were generally much higher than at other European locations, such as Mount Cimone (Italy) [77], Zürich (Switzerland) [78] and Menen (Belgium) [25]. As the distance between Racibórz and the Baltic Sea (over 500 km) precludes the marine origin of Cl and S, the two elements were probably anthropogenic, which was further substantiated by the values of EF (Figure 2). The concentrations of Cl and S in the air in Racibórz, just like in other locations in Silesia, are affected by the intensity of fossil fuel combustion, and more precisely the combustion of hard coal and various solid waste in domestic low-efficiency furnaces [16,70]. This finding was confirmed by the strong seasonal variability in the ambient concentrations of Cl and S and statistically significant difference between the mean concentrations of both these elements between heating and non-heating periods (Table 2). 

Similarly to the PM_2.5_-bound Cl and S, the concentrations of other (perhaps all) elements could be affected by coal and biomass combustion in Racibórz during the heating season. This is indicated by evidently greater values of EF for such elements as compared with the non-heating season (Figure 2), and by definitely greater concentrations of the elements in the heating season. It even applies to those elements which are usually associated with crustal or soil matter (Na, K, Al, Ti, Mg, Si, Ca, and Fe (e.g., [15,16,23]). Although EF does not exceed 10 during the cold season in the case of a majority of such elements, the mere increase in EF in comparison to its values noted during the warm season indicates an influence of anthropogenic emission, e.g., from coal or biomass combustion, on the concentrations of the crustal elements. That is why the origin of PM_2.5_-bound elements in Racibórz is more difficult to interpret (especially in the cold/heating season) than in other regions of Europe [16,22,23,70].

The mean ambient concentrations of most PM_2.5_-bound elements recorded in Racibórz in 2011–2012, just like the mean PM_2.5_ mass concentration, are comparable to the values reported in different cities in southern Poland [15,70,79]. On the other hand, most of the heavy metals were found to have higher concentrations than in other regions of Poland [15,24,70] and Europe [26,61,71,78,80].

The International Agency for Research on Cancer (IARC) of the World Health Organization (WHO) classified As, Cd, Ni and their compounds, as well as Cr(VI) within Group 1 (carcinogenic to humans). The Pb compounds are included in Group 2A (probably carcinogenic to humans). In 2011–2012, the ambient concentrations of As, Cd, Ni, and Pb in Racibórz did not exceed the permissible values of annual concentrations established by the European Commission (6 ng·m^−3^, 5 ng·m^−3^, 20 ng·m^−3^, and 0.5 µg·m^−3^, respectively) [81]. However, they were much higher than elsewhere, e.g., in Flanders (Belgium), Thessaloniki (Greece), Athens (Greece), Bobadela (Spain), Huelva (Spain), Venice (Italy), different sites in Switzerland, Beijing (China) and Rio Grande (Brazil) (Table 3).

### 3.3. Source Apportionment of PM_2.5_

**Figure 4:**
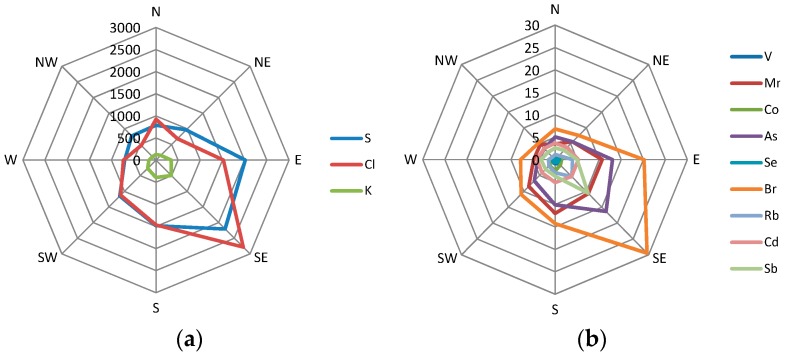

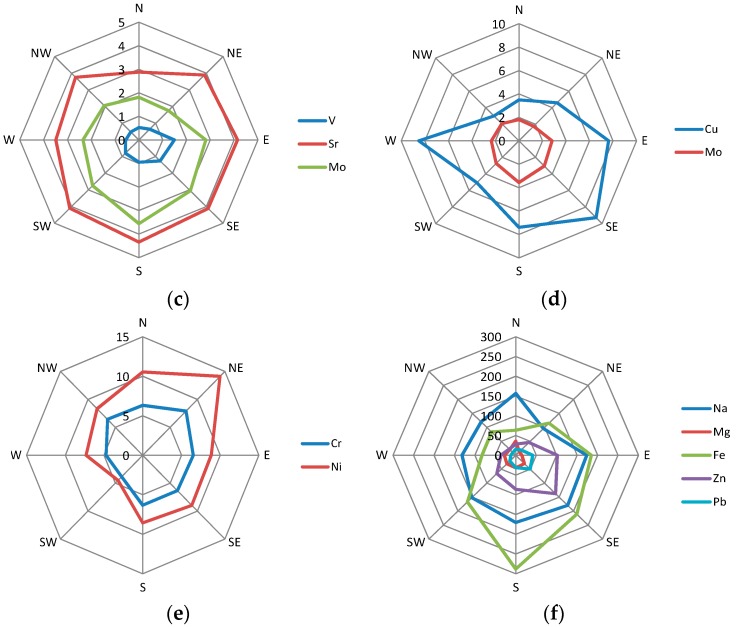
Concentrations (ng·m^−3^) of the selected PM_2.5_-bound elements averaged for 2011–2012 for the selected air mass inflow directions in Racibórz: (**a**) S, Cl, K; (**b**) V, Mn, Co, As, Se, Br, Rb, Cd, Sb; (**c**) V, Sr, Mo; (**d**) Cu, Mo; (**e**) Cr, Ni; (**f**) Na, Mg, Fe, Zn, Pb.

As in the case of the studies listed in Table 1, the authors of this study attempted to identify the sources of PM_2.5_ and the contribution of these sources to the concentrations of PM_2.5_ based on the PCA and MLRA results. Table 3 presents the results of PCA and MLRA modelling performed for the data concerning the elemental composition of PM_2.5_ in Racibórz. For a better interpretation of the PCA results, the enrichment factors (EFs) for PM_2.5_-bound elements (Figure 2) were also taken into account. The inference process also took into consideration the distribution of element concentrations for particular wind directions (Figure 4) and conclusions drawn by the authors of previous studies [21,70], as well as conclusions from available studies by other researchers (Table 1). 

In the PCA model, five new variables—principal components PC1–PC5—were identified. Only the principal components with eigenvalues >1.0 were considered (according to the Kaiser criterion). Their variances were within the range of 4.7%–37.9%. 

Seventeen elements were strongly correlated (factor loadings ≥0.4) with PC1: Zn, K, Br, Rb, As, Pb, Cl, S, Na, Cd, Mn, Co, Se, V, Sb, Mg, and Fe. The mean ambient concentrations of all these elements (except Fe) were statistically significant and evidently higher in the heating (cold) season than in the non-heating (warm) season (Table 2). High concentrations of K, S, Cl, V, Mn, Co, As, Se, Br, Rb, Cd, and Sb were observed on days when there was an air inflow from the S-E and E directions (Figure 4). The local sources of PM_2.5_ (small boiler rooms and individual home furnaces) were located in both directions (Figure 1). The pollutants from the Silesian agglomeration, where the emission from the municipal sector is a dominant source of PM in the heating season [20,70,72,73,87] were transported mainly from the N-E, E, and S-E directions. These observations and conclusions drawn from the previous studies (Table 1; [21,70]) allow us to assume that the PM_2.5_ identified in PC1 originate from the combustion of coal (e.g., the S, As, Br, and Se correlations with PC1), biomass (e.g., the Na and K correlations with PC1) and various waste types (e.g., the Cl correlation with PC1) for heating. This source was assigned with FF_BB_WI symbol, i.e., fossil fuels (FF) and biomass (BB) combustion, and waste incineration (WI; Table 1). 

**Figure 5:**
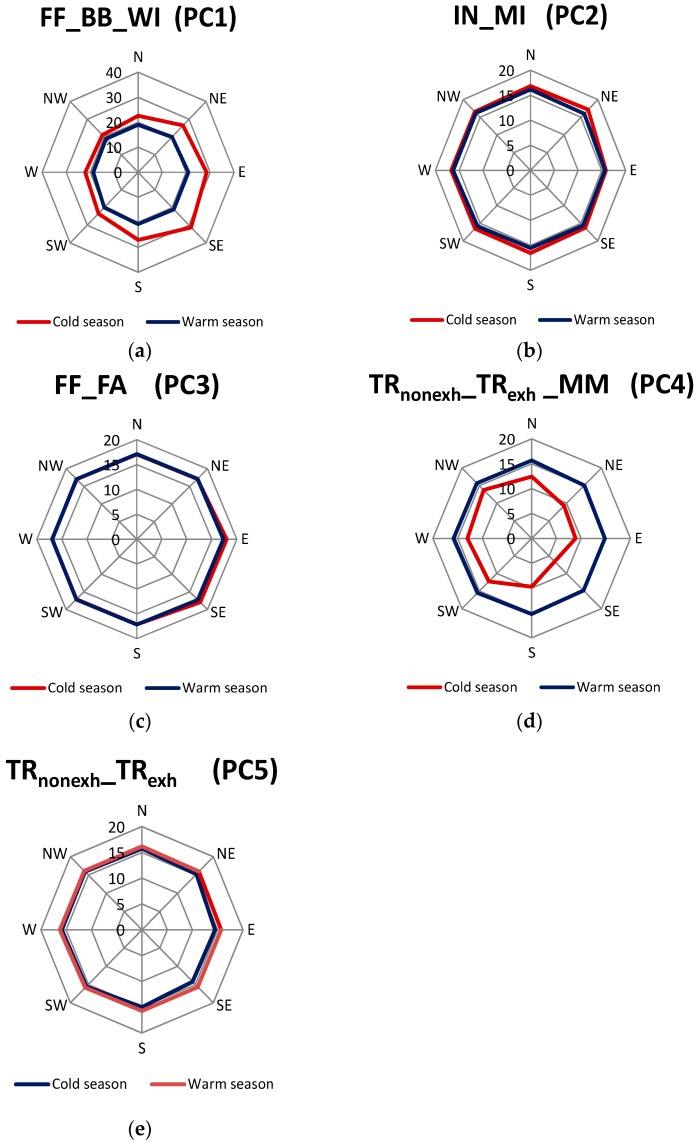
Mean source contributions (%) to PM_2.5_ concentrations in the cold/heating season and the warm/non-heating season for the selected air mass inflow directions in Racibórz. (**a**) Fossil fuel combustion (FF)/biomass burning (BB)/waste incineration (WI); (**b**) industrial sources (IN)/metal industry (MI); (**c**) fossil fuel combustion (FF)/fly ash (FA); (**d**) non-exhaust traffic-related sources (TR_nonexh_)/exhaust traffic-related sources (TR_exh_)/mineral matter (MM); (**e**) TR_nonexh_/TR_exh_.

Mean mass percentages of FF_BB_WI in the PM_2.5_ concentrations in Racibórz were the greatest in the S-E and E directions (about 30%) and slightly smaller in the S and N-E (Figure 5). Generally, much greater mass contents of PC1 (FF_BB_WI) in the PM_2.5_ concentrations occurred during the cold season: the highest twenty-four-hour share of PC1 in the PM_2.5_ concentration, 40.3%, was recorded on 3 February 2012, whereas the lowest share was observed on 11 August 2012 (17.6%). 

The EFs calculated for each of the 17 elements correlated with PC1 were much higher in the heating season than in the non-heating season (Figure 2), which indicates that the PC1 must have been correctly identified as FF_BB_WI. It can therefore be suspected that a stronger anthropogenic influence on the concentrations of the 17 elements during the heating season is a result of a more intensive emission from FF_BB_WI. It must be noted, however, that the mean value of EF for some of the elements, correlated with PC1, i.e., Fe, Mg, Ca, Na, and K, during the non-heating season did not exceed 10, which suggests their natural (crustal) origin. This finding implies that the mineral/crustal matter could have been their source in the warm season. 

PC3 revealed a slightly lower mean percentage (17%) compared to PC1 (Table 4). The variability in its twenty-four-hour percentage in the PM_2.5_ concentrations was low (16.3%–19.2%) in the entire analyzed period. PC3 was the most strongly correlated with Sr, Mo, and V. In 2011–2012, those three elements had statistically higher concentrations in the heating (cold) season (Table 1). Their EFs, although a little higher in the heating season than in the non-heating season, in both seasons were distinctly higher than 10, therefore revealing an anthropogenic origin (Figure 2). The concentrations of Sr, Mo, and V did not differ much for the analyzed air inflow directions (Figure 4). 

The above-mentioned observations allow a conclusion that PC3, similarly to PC1, may have characterized a source of PM_2.5_ whose efficiency changed with air temperature. Its contribution to the concentrations of PM_2.5_ in Racibórz did not depend on the direction of air masses (Figure 5). The energy production based on the combustion of fossil fuels seems to be the source. Widory et al. [88] made observations which definitely confirmed that Sr could be a marker of coal combustion. It was also shown that relatively high concentrations of Mo are present in the fly ash emitted during fossil fuel combustion [89]. Racibórz is under the influence of air masses which are extremely polluted with flue gases emitted by Polish (E and N), Czech (S) and German (W) power and heat and power plants fired with brown and hard coal (Figure 1). According to the symbols used in Table 1, the source identified with PC3 was given the symbol of FF_FA, i.e., fossil fuels (FF) and fly ash (FA).

Cu and Mo were the most strongly correlated with PC5. Their concentrations were the highest at the inflow of air masses from the center of Racibórz and other cities of the Silesian agglomeration (S-E and E). High concentrations of these elements were also observed at the inflow of air masses from the Czech Republic and Germany (S, W), especially from the Moravian-Silesian Region (S; Figure 4). Cu and Mo demonstrated statistically higher mean concentrations in the heating season than in the non-heating one (Table 2). The higher EF values of Cu and Mo were observed in the heating season (Figure 2). Therefore, in Racibórz, both Cu and Mo could have partially originated from fuel combustion (PC1, PC3). However, most authors treat PM-bound Cu as the marker of traffic emission (Table 1). Mo is present in the gases coming from the combustion of fossil fuels, which includes petrol and crude oil [90,91]. In 2011–2012, distinct differences in the twenty-four-hour percentage of PC5 in PM_2.5_ were found in Racibórz (1.0%–16.5%). The lowest percentage of PC5 in PM_2.5_ was observed on cold (heating) season days. The values for the days analyzed in the warm season were much higher. Generally, a mean mass content of PC5 in the concentrations of PM_2.5_ did not depend on the direction of the incoming air masses (Figure 5). This finding supported the assumption that that PC5 characterized emissions from traffic (given the symbol of TR_nonexh__TR_exh_). PC4 was classified in the same way. It was the most strongly correlated with Co and Al, which suggested that tire and road wear particles were some of the identified sources of PM_2.5_ in Racibórz [92]. Yet, in the case of PC4, a clearly greater mean mass content in the concentration of PM_2.5_ occurred during the warm season rather than in the cold part of the year. Besides, the greatest percentages of PC4 in PM_2.5_ were determined for the western and northern directions (Figure 5), where the measuring site was surrounded by farmland and meadows. Eventually, it was decided that PC4 may represent, apart from traffic emissions, also soil matter; it was assigned the symbol of TR_nonexh__TR_exh__MM. The percentage of the mineral/soil matter (exclusively) in the PM_2.5_ mass in Racibórz was calculated in a simplified way in the previous study [70]. Its mean mass percentage did not exceed 3.5% in the entire measurement period. 

The percentage of the last extracted principal component (PC2) was independent of the direction of incoming air masses or the season (Figure 5). Cr and Ni (i.e., two out of three elements whose EFs did not differ between the heating season and the non-heating season, Figure 2) were the most strongly correlated with PC2. Their mean ambient concentrations were higher in the non-heating season, therefore they could not have come from the production of energy for heating. Sc was the third element strongly correlated with PC2. The results presented in Table 1 clearly indicate that PC2 (due to its strong correlation with Cr) reflects the industrial source of PM_2.5_ in Racibórz (most probably steel smelter emissions; according to the symbols used in Table 1, the source identified with PC2 was given the symbol of IN_MI, i.e., industrial sources (IN) and metal industry (MI)). Cr is not the only marker of this source. Also Ni is a common ingredient in steel and other metal products [93,94]. Other elements correlated with PC2 (e.g., Si, Mg, Na, Ca, Ti) are either the ingredients of the fluxes introducing the necessary components into the steel processing, such as components for the necessary composition (e.g., CaO, CaCO_3_, SiO_2_, Al_2_O_3_, or CaF_2_), or oxidants used for oxidizing the metal stock admixtures and transforming them into slag (such as Fe or Mn ores). Sc and other rare earth elements are broadly used in metallurgy as alloying additions improving the properties of admixture metals, as permanent magnets or polishing pastes. In the case of a majority of elements correlated with PC2, relatively high concentrations (visibly higher for Cr and Ni) were observed when the air masses were transported from the N-E, where the most industrialized cities of the biggest urban and industrial center of Poland (Upper Silesian Industrial Region) are located, and in the S direction (Moravian–Silesian Region), where the biggest European steel works is situated (ArcelorMittal Ostrava)—Figure 1 and Figure 4. Since there are a number of large steelworks, non-ferrous metal works and other industrial plants in Upper Silesian cities, it is difficult to point at *the* one dominant industrial source in the N-E direction. 

## 4. Conclusions

Southern Poland, where Racibórz is located, is one of the most urbanized and polluted “hot spot” areas in Europe. The PM_2.5_ found at the quasi-rural site in that area differs significantly from the PM_2.5_ at other similar areas located in various parts of Europe. Differences are observed in both the concentrations and elemental compositions of PM. The obtained results indicate that sources originate from the common use of solid fuels for energy production in Poland. Nearly 17% of the PM_2.5_ in the air in Racibórz comes from the hard and brown coal combustion in power plants and large industrial heat and power plants located in various parts of Poland and abroad. In our study we found that 22% of the PM_2.5_ is associated with the combustion of the coal and biomass mixture (and most probably home waste) at local small boiler rooms and home furnaces. In other European regions, traffic emissions are currently the primary source of air pollution (also with PM). In Racibórz, the contribution of traffic related pollutants to the concentration of PM_2.5_ does not exceed 30%. Considering the elemental composition of PM_2.5_ alone is insufficient to determine whether the majority of emissions comprise PM particles of mechanical origin (non-exhaust emission) or particles/particle precursors from fuel combustion (exhaust emission). In southern Poland, the industrial emissions exert a significant effect on PM_2.5_ concentrations. Steelworks, non-ferrous metal works and other industrial plants located outside the Racibórz area, (PC2; jointly referred-to as IN_MI: industrial sources_metal industry) are responsible for 16% of the PM_2.5_ mass in Racibórz.

Long-distance transport of PM is known to be associated with adverse health effects and impacts related to climate change. Source identification of PM-bound elements via principal component analysis and monitoring of PM concentrations are critical tools used to evaluate their potential impacts and effectiveness of control measures. Regionally in Poland and locally in Raciborz the combustion of solid fuels has been identified as primary anthropogenic contributors to air pollution. While total elimination of many anthropogenic sources is not possible, important findings of this study can be used to help inform the development of realistic management policies and cost-effective methods to improve air quality. It is our opinion that simply reducing the combustion of coal, biomass, and wastes in domestic ovens in the Raciborz region would benefit local and regional air quality.

## Figures and Tables

**Table 1:** Particulate matter (PM) source apportionment results based on the principal component analysis (PCA) and multi-linear regression analysis (MLRA) analysis obtained from research conducted at various locations all over the world.

Location, Station Type, Measurement Periods, PM Fraction	Factors Identified Together with Elements Included and Suggested Source Names ^1^						
Diabla Góra (PL ^2^), regional background (EMEP), January–March 2009, PM_10_ [22] ^3,4^	As, Cd, Ni, Pb, Zn, SO_4_^2−^, HNO_3_ + NO_3_^−^ (MI, FA)	Cu, Ni, Zn, SO_4_^2−^, NH_3_ + NH_4_^+^ (SA, (L)RT)	Cr, Cu (AN, TR_exh_, TR_nonexh_)				
Warsaw (PL), urban, November–December 2013, PM_2.5_ [15]	Sc, Se, As, Pb, Br, Mg, K, Zn, Fe, S, Cl, Na, Ca, Co, Sr, Al, Mn, Si, Cu (FF, BB)	Ni, V, Mn, Ca, Si, Cu, Ti (Oil)	Cr, Sr, S (TR_exh_, TR_nonexh_)	Ti, Ni, Al, Si, Cd (MM, TR_nonexh_)			
Wrocław (PL), residential, January–April 2009, PM_2.5_ [23]	H, Cl, K, Ca, Cu, Zn, Br, Rb, Fe, Pb (BB, TR_exh_, TR_nonexh_)	Al, Si, Ti, Ca (MM)	V, Ni, S (Oil)	Cr, Mn, As, Pb, Se, Br, Fe (MI, FF)			
Zabrze (PL), urban background, 2007, PM_2.5_ [16]	Cl, Mn, Fe, Cu, Zn, Br, Pb (TR_exh_, TR_nonexh_, AN)	S, Ca, Ti, Sb (FF, TR_exh_, TR_nonexh_)	Al, K, Sr (MM, BB)				
Katowice (PL), urban background, 2007, PM_2.5_ [16]	S, Cl, K, Cu, Zn, Br, Sb, Pb (TR_exh_, TR_nonexh_, AN)	Ti, Cr, Mn, Fe (MI)	S, Ca, Se (FF)				
Brzezina (PL), rural, August 2009, PM_10_ [24]	K, Ca, Ti, Mn, Fe, Zn, Br, Pb (MM, TR_exh_, TR_nonexh_)	Cu, As (MI_nonfer_)					
Brzezina (PL), rural, February 2010, PM_10_ [24]	K, Ca, Cr, Mn, Fe, Cu, Zn, Br, Pb (AN)	Ca, As (MI_nonfer_)					
Krakow (PL), urban, June 2009, PM_10_ [24]	K, Ca, Mn, Fe, Zn (MI)	Cu, Br, As (TR_exh_, TR_nonexh_)	Ti, Cr (MM)				
Krakow (PL), urban, January 2010, PM_10_ [24]	K, Cu, Zn, As (AN, TR_exh_, TR_nonexh_)	Ca, Cr, Mn, Fe, Br (MI)					
Menen (BE), suburban, 2003, PM_2.5_ [25]	S, Si, Al, K, Ti, Ca, Fe (MM, TR_nonexh_)	Cr, Cu, Zn (TR_exh_, TR_nonexh_)	V, Mn, Ni, Pb (IN)	Br, Rb (IN/MM)			
K-Puszta (HU), regional background (EMEP station), May–June 2006, PM_10_ [26]	Mg, Al, Si, P, K, Ca, Ti, Mn, Fe (MM)	NH_4_^+^, SO_4_^2−^, S, Pb (SA, AN)	EC, Cu (TR_exh_, TR_nonexh_)	Na (SS)	NO_3_^−^ (SA)	Cl (SS)	Zn (AN)
Belgrade (CS), urban background, June 2003–July 2005, PM_2.5_ [27]	Zn, Mn, Fe, Al (TR_nonexh_)	Pb, Cr (TR_exh_, TR_nonexh_, IN)	Ni, V (Oil)	Cu, Cd (TR_exh_, IN)			
Belgrade (CS), urban background, June 2003–July 2005, PM_10_ [27]	Zn, Mn, Fe, Al (TR_nonexh_)	Ni, V (Oil)	Cu, Cd, Pb (TR_exh_, TR_nonexh_)	Pb, Cr (TR_exh_)			
Milan (IT), urban background, 2001, PM_10_ [28]	Al, Si, Ca, Ti, Mn, Fe (MM)	K, Mn, Fe, Cu, Zn, Br, Pb (TR_exh_, TR_nonexh_)	S, K (SA)	Mn, Zn (IN, MI)			
Venice Lagoon (IT), industrial-urban, March 2002–July 2003, PM_3.0_ [29]	Cd, Cu, K, Mn, Ni, Pb, V, Zn (TR_exh_, TR_nonexh_, IN)	Al, Co, Cu, Fe, Mg, Mn, Sr (MM)	Na (SS)				
Llodio (ES), urban background, January–December 2001, PM_2.5_ [30]	Pb, Zn, Cd, Mn, Fe, Cu (MI)	SO_4_^2−^, NH_4_^+^, V, Na, K, Tl ((L)RT, AN)	Al_2_O_3_, Ti, Ba, Ca, Sr (MM)	P, OC, EC, K, Tl, NO_3_^−^ (TR_exh_)	Cr, Ni, Mo, Co, As, Cu, Fe, OC, EC (IN)		
Barcelona (ES), urban background, March–November 2007, PM_1_ [31]	K, Li, Cu, Zn, Ga, Rb, Fe, Ti, Mn, Sr, Sb, Ba (MM)	TC, NO_3_^−^, Cl, As, Se, Cd, Sn, W, Pb, Mn, Sb (TR_exh_, TR_nonexh_)	Al_2_O_3_, Ca, Na, Mg, La, Ce, Ti, Sr (MM)	SO_4_^2−^, NH_4_^+^, V, Co, Ni (Oil)			
Barcelona (ES), urban background, March–November 2007, PM_2.5_ [31]	Al_2_O_3_, Ca, K, Mg, Fe, Li, Ti, Ga, Rb, Sr, La, Ce, Mn, Co, P (MM)	TC, NO_3_^−^, Cu, As, Se, Cd, Sn, Sb, W, Pb, Bi, Mn, Fe, P, Zn (TR_exh_, TR_nonexh_)	NH_4_^+^, V, Ni, Ba, Co (Oil)				
L’Hospitalet (ES), urban-kerbside, June 1999–June 2000, PM_2.5_ [32]	Fe, K, Mn, Pb, Zn, Cu, Cr, Ni, V, OC + EC, Cl, NO_3_^−^, NH_4_^+^ (TR_exh_, TR_nonexh_)	Ca, Al_2_O_3_, Fe, Mg, Ti, Sr, K, Mn (MM)	K, Ni, V, nss-SO_4_^2−^, NO_3_^−^, NH_4_^+^ (FA)	Al_2_O_3_, P, Na, NO_3_^−^ (IN)			
Monagrega (ES), rural, March 1999–July 2000, PM_10_ [32]	Ca, Al_2_O_3_, Fe, Mg, Ti, Sr, K, Mn, V (MM)	Pb, Zn, V, OC + EC, nss-SO_4_^2−^, NH_4_^+^ (FA)	Mg, Na, Cl (SS)	Pb, OC + EC, NO_3_^−^ (TR_exh_, TR_nonexh_)			
Santa Ana (ES), suburban, January 2004–March 2005, PM_2.5_ [33]	Ti, Fe, Al_2_O_3_, Mn, Rb, K, Ca (MM)	OM, EC, NO_3_^−^, Cl, Sb, K, Pb, As, NH_4_^+^, Mg (TR_exh_, TR_nonexh_)	SO_4_^2−^, V, Ni, As, NH_4_^+^ (SA, IN)	Na, Cl (SS)	Zn (MI)		
Huelva (ES), urban, April 2008–December 2009, PM_2.5_ [34]	nss-SO_4_^2−^, NO_3_^−^, NH_4_^+^, P, As, Pb, Cd, V, Ni, Zn, Bi, Mo, Sn (IN)	OM, Al, Ca, Fe, Ti, Mn, K (TR_exh_, TR_nonexh_)	Na, Cl^−^, Mg (SS)				
Lisbon (PT), suburban-industrial, 2001, PM_2.5_ [35]	Al, Si, Sc, Ti, Mn, Fe, La, Sm, Ca^2+^ (MM)	V, Ni, Co, Pb (Oil)	Cl^−^, Na^+^, Mg^2+^, Br (SS)	Se, Hg (IN)	SO_4_^2−^, NH_4_^+^, Cl^−^ (SA)	Cu, Zn, Sb, Pb (IN, TR_exh_, TR_nonexh_)	As, NO_3_^−^, K^+^, NH_4_^+^ (TR_exh_)
Izmir (TR), suburban, June 2004–May 2005; PM_2.5_ [36]	Ba, Ca, Fe, Mg, Sr (MM)	Cd, Mn, Pb, V, Zn (MI, FF)	Al, Cu (TR_exh_, TR_nonexh_)	K, Na (SS)			
Bishkek and Teplyklouchenka (KG), remote sites, July 2008–July 2009, PM_2.5_ [37]	Cl, Li, B, Na, Mg, Al, P, K, Ca, Sc, Ti, V, Cr, Mn, Fe, Co, Rh, Sr, Y, Nb, Pd, Cs, Ba, La, Pr, Nd, Sm, Eu, Gd, Ho, Tm, Yb, Lu, W, Th, U (MM)	Cu, Zn, Rh, Pd, Pb (MI, AN)	OC, EC, SO_4_^2−^, NO_3_^−^, NH_4_^+^, Sb (SA)	Mn, Cd, As, Tl (AN)			
Jorhat City (IN), urban, January 2007–January 2008, PM_2.5_ [38]	Al, Si, Ca, Ti (MM)	S, SO_4_^2−^, Te, Mn, Cd, Sn, Sb (FF)	Co, Ni, Cu, Zn, Cd, Te (IN, TR_exh_, TR_nonexh_)	K, NH_4_^+^ (BB)	NO_3_^−^, NH_4_^+^, SO_4_^2−^ (SA)		
Kanpur City (IN), residential, July 2008–May 2009, PM_1_ [39]	Cu, Zn, Pb (TR_exh_, TR_nonexh_)	Ca, Mg, Zn, Cr, Fe, Pb, V (TR_nonexh_)	NO_3_^−^, SO_4_^2−^ (SA)	Cl^−^, Se, Cd, Pb, Ni (FF)			
Agra (IN), rural, May 2006–March 2008, PM_2.5_ [40]	Pb, Ni, Zn, Cu (IN, (L)RT)	Ni, Fe, Cr (MM)	Cr, Mn (MM, TR_exh_, TR_nonexh_)				
Agra (IN), urban, May 2006–March 2008, PM_2.5_ [40]	Zn, Cr, Cu (IN)	Pb, Ni, Mn (TR_exh_, TR_nonexh_)	Ni, Fe (WI, WD)				
Ordos (CN), urban, September 2005, PM_2.5_ [41]	Al, Ca, Fe, Mg, Mn, Na, P, Sr, Ti (MM)	B, Ba, Ca, Na, Sr, Cl^−^, OC, EC (TR_exh_)	K, Pb, Zn, NO_3_^−^, SO_4_^2−^, OC, EC (SA)	Cr, Cu, Ni (IN, Oil)			
Xinglong (CN), rural mountainous site, September 2008, PM_2.5_ [42]	Na, Mg, Al, K, Ca, Cr, Mn, Fe, Ni, As, Mo, Ba, U (MM, FA)	K, Zn, Ag, Cd, Tl, Pb (IN, TR_exh_, TR_nonexh_, BB)	Be, Al, Mo, Ag, Cd, Th (MM)	Cr, Cu, Se (MI_nonfer_)	Co, Sb (TR_nonexh_)		
Beijing (CN), urban, 2000, PM_2.5_ [43]	Al, Si, Ca, Ti, Fe, Mg (TR_nonexh_)	EC, Mn, Cu, Zn, As, Pb (TR_exh_)	OC, NO_3_^−^, Cl, K, Br (FF, BB)	NO_3_^−^, SO_4_^2−^, NH_4_^+^, As (SA)	Ni, Se (IN, e.g., MI)		
Beijing (CN), roadside, 2008–2009, PM_1.0–2.5_ [44]	Al, Ti, Mg, Si, Ca, Na, K, Fe, Mn, Br, Cl, Cu (MM, TR_nonexh_)	Cl, Cu, Zn, Pb (IN, FF)	Br, NH_4_^+^, NO_3_^−^, SO_4_^2−^ (SA)				
Ji’nan (CN), urban, September 2010, PM_2.5_ [45]	Cu, Fe, Mn, Ni, Pb, Sr, Zn (TR_exh_, TR_nonexh_, MI, FF)	Ba, Ni, Sr, Ti (MM, TR_nonexh_)	As, Cr (FF)				
Chengdu (CN), urban, April 2009–January 2010, PM_2.5_ [46]	NH_4_^+^, K^+^, Cl^−^, NO_3_^−^, SO_4_^2−^, OC, EC, Cr, Zn, As, Br, Pb, Cu, Mn, Rb, Mo (AN)	Al, Si, Ca, Ti, Fe, Mn, Ba (MM)	Na, Mg^2+^, Ca^2+^ (MM)	Sr, Cd (MI)			
Changsha (CN), suburban, July and October 2008, PM_10_ [47]	Al, Si, Ti, Mg, Fe, Cl, Ca, Na (MM)	Zn, Pb (TR_exh_, TR_nonexh_)	S, P, K (FF, SA)	Mn, K, Ca, Na (BB, WI)	Ni (Oil)	Cu (IN)	
Lhasa (CN), urban, September 2007–August 2008, PM_10_ [48]	Na, Mg, Al, K, Ca, Sc, Ti, V, Mn, Fe, As, Ba, Pb ((L)RT, MM, TR_nonexh_)	Na, Ni, Cu, Zn, As, Pb (AN)	V, Cr, Co, As, Cd (WI)				
Tunghai University (TW), rural, July 2001–April 2002, PM_2.5_ [49]	Fe, Mg, Cd (MM)	Pb (TR_exh_, TR_nonexh_)	Cr, Cu (MI)				
Jeongwang (KR), residential, May 2004–January 2006, PM_10_ [50]	Al, Ba, Cr, Fe, K, Na, Sb, Ti, V (MM, TR_nonexh_)	Al, As, Cd, Mn, Ni, Pb, Se, V, Zn (AN)	As, Cr, Pb, Sb, Se, V, Zn (IN)	Cd, Pb, Sb, V (IN)	K, Na (SS)		
Yeongwoi (KR), urban, April 2012–October 2013, PM_2.5_ [51]	Al, Si, K, Ca, Mn, Fe, As, Pb (MM)	Cr, Ni (IN)	Zn, Cd (IN)				
Pohang (KR), residential, 2003–2004, PM_10_ [52]	Ba, Cd, Co, Fe, Mn, Ni, Pb, Sb, V, Zn (WI)	Al, Ca, Co, Fe, Mn, Si, Ti (MM)	K, Mg, Na (SS)	Cr, Ni (FF, Oil)	Cu, Tl (IN, MI)		
NAPS network sampling sites (CA), urban and rural sites, May 2004–December 2006, PM_2.5_ [53]	Mn, Fe, Zn, Mo, Sb (MM, TR_nonexh_)	Se, Sn, Cd, Pb (FF)	V, Ni (IN, Oil)	Sr, Ba, Sb (TR_nonexh_)	As, Cu, Sb, Cd, Zn (IN)		
Los Angeles (USA), 10 sampling sites, April 2008–March 2009, PM_0.25_ [54]	Rb, Mg, Al, K, Mn, Ca, Ti, Na, Li, Fe, Sr, Co (TR_exh_, TR_nonexh_)	Fe, Sr, Rh, Ba, Sb, Cu, Mo, As, Pb (TR_nonexh_)	S, La, V (Oil)	Cd, Ag, Pb (MI)	Cr, Ni (MI)		
Rio de Janeiro (BR), different locations, September 2003–December 2005, PM_2.5_ [55]	Al, Fe, Ce (MM)	BC, Cu, Cd (TR_exh_, TR_nonexh_)	Ni, V, SO_4_^2−^ (Oil, SA)	Na, Mg (SS)			
Buenos Aires (AR), urban, October 2005–October 2006, PM_10_ [56]	Sc, Sm, Ce, Fe, Cs, Cr (MM)	Fe, BC, Zn, As, Ba, Sb (TR_exh_, TR_nonexh_)	Zn, Br, Sb (MI_nonfer_, WI)	Eu, Co, La (AN)	Na (SS)		

^1^ PM sources: AN—unidentified anthropogenic sources; BB—biomass burning; FA—fly ash; FF—fossil fuel combustion; IN—industrial sources (different, e.g., from cement industry, pigment manufacture plant, petrochemical industry, oil refineries); (L)RT—regional and/or long-range transport; MM—mineral matter (e.g., crustal/soil dust, construction dust, mechanical abrasion processes of crustal materials, soil-related industry, etc.); MI—metal industry; MI_nonfer_—non-ferrous metal processes; Oil—fuel oil combustion; SA—secondary aerosol compounds; SS—sea salt; TR_exh_—exhaust traffic-related sources; TR_nonexh_—non-exhaust traffic-related sources; WD—waste dumping; WI—waste incineration. ^2^ ISO 3166 country codes: AR—Argentina; BE—Belgium; BR—Brazil; CA—Canada; CN—China; CS—Serbia and Montenegro; ES—Spain; HU—Hungary; IE—Ireland; IN—India; IT—Italy; KG—Kyrgyzstan; KR—South Korea; NZ—New Zealand; PL—Poland; PT—Portugal; TR—Turkey; TW—Taiwan; USA—United States of America. ^3^ References. ^4^ PM_10_—particles with aerodynamic diameters ≤10 µm.

**Table 2:** Descriptive statistics of twenty-four-hour concentrations of PM_2.5_ (µg·m^−3^) and PM_2.5_-bound elements (ng·m^−3^) in the warm and the cold seasons of 2011–2012 in Racibórz.

Element	Cold/Heating Season (*n* = 147)			Warm/Non-Heating Season (*n* = 173)			Concentration Ratio ^1^
	Mean ± SD ^2^	Min	Max	Mean ± SD	Min	Max	
PM_2.5_	48.7 ± 39.4	3.6	209.5	13.9 ± 8.0	3.2	42.6	**3.5**
Na	201.3 ± 109.1	-	644.5	95.0 ± 46.6	-	237.5	**2.1**
Mg	44.6 ± 35.6	-	201.7	10.3 ± 13.7	-	49.1	**4.3**
Al	37.5 ± 33.7	-	198.1	47.4 ± 250.5	-	3296.4	**0.8**
Si	73.0 ± 63.4	12.4	363.3	163.8 ± 148.0	28.2	1073.0	**0.5**
S	1528.0 ± 1152.2	186.1	6812.9	924.4 ± 399.3	243.1	2283.5	**1.6**
Cl	2364.3 ± 2274.3	24.1	13,801.1	206.3 ± 279.3	15.2	1525.3	**11.5**
K	384.8 ± 307.4	38.5	2360.8	138.0 ± 89.6	20.9	505.3	**2.8**
Ca	56.3 ± 41.7	8.9	277.4	71.3 ± 64.7	7.9	678.9	**0.8**
Cs	3.3 ± 2.8	-	18.8	7.5 ± 11.8	0.5	145.0	**0.4**
Ti	2.4 ± 1.9	-	9.4	3.7 ± 3.8	0.2	28.3	**0.6**
V	0.9 ± 0.8	0.2	4.4	0.7 ± 0.8	-	5.3	**1.3**
Cr	3.6 ± 5.5	0.5	38.3	7.4 ± 7.2	0.5	45.4	**0.5**
Mn	8.0 ± 6.6	-	41.2	7.0 ± 4.1	1.4	19.3	1.1
Fe	135.5 ± 136.7	10.9	722.9	159.1 ± 114.7	15.6	496.8	**0.8**
Co	1.6 ± 2.0	-	10.8	0.9 ± 1.4	-	6.2	1.8
Ni	5.5 ± 9.3	0.2	55.8	10.8 ± 12.1	0.2	85.0	0.5
Cu	9.3 ± 24.1	1.0	289.3	3.8 ± 1.8	1.0	13.5	**2.5**
Zn	99.9 ± 90.1	6.5	544.4	32.9 ± 27.3	2.7	158.6	**3.0**
As	11.3 ± 11.5	0.5	70.9	4.2 ± 2.8	0.2	15.0	**2.9**
Se	0.8 ± 1.1	-	6.9	0.3 ± 0.5	-	2.2	**2.3**
Br	21.6 ± 20.0	2.9	111.6	4.3 ± 2.9	0.5	17.4	**5.1**
Rb	3.9 ± 3.9	0.2	22.6	0.9 ± 0.6	-	3.6	**4.5**
Sr	4.3 ± 2.5	0.5	18.8	3.5 ± 1.7	0.5	14.0	**1.2**
Mo	3.0 ± 1.9	-	12.6	2.5 ± 3.5	-	44.5	**1.2**
Cd	5.2 ± 2.5	1.4	17.3	3.7 ± 1.2	1.4	7.0	**1.4**
Sb	6.0 ± 8.1	0.2	58.0	2.8 ± 1.9	-	10.9	**2.2**
Ba	5.1 ± 3.4	-	22.6	5.3 ± 4.1	-	48.8	0.9
Pb	34.0 ± 34.2	-	204.9	13.5 ± 9.2	-	54.5	**2.5**

^1^ ratio between the mean concentration in the cold/heating season (January–March and October–December) and the mean concentration in the warm/non-heating season (April–September); underlined values in bold indicate that the difference between the concentrations observed in the cold and the warm periods is statistically significant—non-parametric Mann-Whitney U test, α = 0.05). ^2^ SD—standard deviation.

**Table 3:** The mean concentrations of PM-related As, Cd, Ni, and Pb at various sites in the world.

Location City (Country), Site Type	Measurement Period	PM Fraction	Concentrations (ng·m^−3^)			
			As	Cd	Ni	Pb
Racibórz (Poland), suburban (this study) ^a^	January 2011–December 2012	PM_2.5_	11.3/4.2	5.2/3.7	5.5/10.8	34.0/13.5
Flanders (Belgium), suburban [81]	September 2006–September 2007	PM_10_	3.8	-	3.6	21.0
Thessaloniki (Greece), residential-commercial [82] ^a^	June 1994–May 1995	PM_2.5_	1.5/1.4	0.71/1.3	15/21	122/141
		PM_2.5–10_	0.59/0.47	0.12/0.10	6.4/5.2	29/29
Athens (Greece), suburban [83]	August–November 2003	PM_2.5_	5.78	0.58	2.19	10.4
Bobadela (Spain), suburban-industrial [35]	2001	PM_2.5_	0.31	-	2.6	8.6
		PM_2.5–10_	0.16	-	1.6	6.0
Huelva (Spain), urban background [34]	April 2008–December 2009	PM_2.5_	5.1	0.6	2.3	10.8
		PM_2.5–10_	1.1	0.1	1.4	3.6
Venice (Italy), urban-industrial [29]	March 2002–June 2003	PM_10_	3.0	2.0	14	19
Zurich-Kaserne (Switzerland), urban [84]	April 1998–March 1999	PM_2.5_	0.47	0.31	3.1	21
		PM_2.5–10_	0.10	0.03	0.11	5.9
Basel (Switzerland), suburban [84]	April 1998–March 1999	PM_2.5_	0.40	0.48	1.7	19
		PM_2.5–10_	0.11	0.04	0.46	4.4
Chaumont (Switzerland), rural [84]	April 1998–March 1999	PM_2.5_	0.16	0.12	1.3	4.7
		PM_2.5–10_	0.02	1.00	0.04	0.8
Beijing (China), urban [85]	February 2005–September 2007	PM_2.5_	13	2.5	1.6	32
Rio Grande (Brazil), urban-industrial [86] ^b^	October 2009–January 2010	PM_2.5_	bld.	bld.	0.79/0.86	0.40/bld.

^a^ Mean concentrations of the elements are shown separately for cold period/warm period; ^b^ mean concentrations of the elements are shown separately for October 2009/January 2010; bld.—below limit of detection.

**Table 4:** Results of the principal component (PCA) and multi-linear regression (MLRA) analyses performed for PM_2.5_ and PM_2.5_-bound element concentrations.

Component	Element Factor Loading ^1^	Source/%Variance	Mean Source Contributions (%) to PM_2.5_ Concentrations in Sampling Period (Results from MLRA) ^2^
PC1	Zn_0.96_, K_0.95_, Br_0.93_, Rb_0.94_, As_0.91_, Pb_0.90_, Cl_0.87_, S_0.82_, Na_0.69_, Cd_0.65_, Mn_0.64_, Co_0.64_, Se_0.60_, V_0.58_, Sb_0.54_, Mg_0.53,_ Fe_0.52_	FF_BB_WI ^3^/37.9	22.0
PC2	Cr_0.86_, Ni_0.84_, Si_0.71_, Mg_0.70_, Na_0.62_, Sc_0.58_, Ca_0.55_, Ti_0.42_, Ba_0.42_	IN_MI ^4^/19.4	16.2
PC3	Sr_0.63_, Mo_0.49_, V_0.43_	FF_FA ^5^/10.1	17.2
PC4	Co_0.47_, Al_0.42_	TR_nonexh__TR_exh__MM ^6^/7.1	13.5
PC5	Cu_0.85_, Mo_0.46_	TR_nonexh__TR_exh_/4.7	15.7
	Total variance	79.3	84.6%

^1^ Elements with factor loadings <0.40 are not included. Elements presented in the descending order of their factor loads, with factor loadings indicated as subscript. ^2^ Sets of measured 24-h PM_2.5_ concentrations and concentrations computed for each day from MLRA—the determined contributions were substantially correlated (*R*^2^ = 0.86). ^3^ FF_BB_WI—PM_2.5_ sources: FF—fossil fuel combustion; BB—biomass burning; WI—waste incineration. ^4^ IN_MI—PM_2.5_ sources: IN—industrial sources; MI—metal industry. ^5^ FF_FA—PM_2.5_ sources: FF—fossil fuel combustion; FA—fly ash. ^6^ TR_nonexh__TR_exh_ _MM—PM_2.5_ sources: TR_nonexh_—non-exhaust traffic-related sources; TR_exh_—exhaust traffic-related sources; MM—mineral matter (e.g., crustal/soil dust, construction dust, mechanical abrasion processes of crustal materials, soil-related industry, etc.).
